# Radiation Doses in Cardiovascular Computed Tomography

**DOI:** 10.3390/life13040990

**Published:** 2023-04-11

**Authors:** Bartłomiej Kędzierski, Piotr Macek, Barbara Dziadkowiec-Macek, Krystian Truszkiewicz, Rafał Poręba, Paweł Gać

**Affiliations:** 1 Department of Radiology and Imaging Diagnostics, Emergency Medicine Center, Marciniak Lower Silesian Specialist Hospital, Fieldorfa 2, 54-049 Wrocław, Poland; 2 Department of Internal Medicine, Occupational Diseases, Hypertension and Clinical Oncology, Wroclaw Medical University, Borowska 213, 50-556 Wrocław, Poland; 3 Department of Population Health, Division of Environmental Health and Occupational Medicine, Wroclaw Medical University, Mikulicza-Radeckiego 7, 50-368 Wrocław, Poland

**Keywords:** computed tomography, cardiovascular system, radiation dose, TAVI procedures

## Abstract

We discussed the contemporary views on the effects of ionising radiation on living organisms and the process of estimating radiation doses in CT examinations and the definitions of the CTDI, CTDIvol, DLP, SSDE, ED. We reviewed the reports from large analyses on the radiation doses in CT examinations of the coronary arteries prior to TAVI procedures, including the CRESCENT, PROTECTION, German Cardiac CT Registry studies. These studies were carried out over the last 10 years and can help confront the daily practice of performing cardiovascular CT examinations in most centres. The reference dose levels for these examinations were also collected. The methods to optimise the radiation dose included tube voltage reduction, ECG-monitored tube current modulation, iterative and deep learning reconstruction techniques, a reduction in the scan range, prospective study protocols, automatic exposure control, heart rate control, rational use of the calcium score, multi-slices and dual-source and wide-field tomography. We also present the studies that indicated the need to raise the organ conversion factor for cardiovascular studies from the 0.014–0.017 mSv/mGy*cm used for chest studies to date to a value of 0.0264–0.03 mSv/mGy*cm.

## 1. Introduction

Fifty years have passed since the invention of computed tomography by Sir Godfrey Hounsfield in 1972. During this time, this method has revolutionised imaging diagnostics in medicine [1]. The first application of computed tomography with ECG acquisition monitoring was implemented by George Harell and Diana Guthaner from Stanford University [2] in 1978, and the images of the heart obtained by this team, including the coronary vessels in computed tomography, were published in 1979 [3]. Today we would judge these images as very imperfect, as they were created using sequential computed tomography. The extensive use of computed tomography in heart diagnostics became possible only after the introduction of the helical acquisition method and the improvement of the temporal and spatial resolution of CT machines in the following years. In 1992, the images from the one-slice camera were published. In 1994, a retrospective reconstruction technique was developed from the ECG-gated studies. In 1999, four-slice computed tomography was introduced, followed by 16-slice in 2002 [4,5].

Currently, cardiac computed tomography is part of the guidelines of the European Society of Cardiology and repeated by the Polish Society of Cardiology. In the diagnosis of chronic coronary syndromes, CT is recommended as an initial test for the diagnosis of coronary artery disease for symptomatic patients when coronary artery disease with stenoses in the coronary arteries cannot be ruled out by clinical assessment alone. It is recommended that CT be considered as an alternative to invasive coronary angiography (ICA) if the results of the other non-invasive tests are inconclusive or non-diagnostic [6]. On the other hand, in the diagnosis of acute coronary syndromes, CT is recommended as an alternative to ICA to exclude the acute coronary syndrome if the probability of coronary artery disease is low or moderate and when the cardiac troponin levels and/or ECG results are normal or inconclusive [7].

As a result of significant technological development of the apparatus, cardiac CT achieves a temporal resolution of 76 ms and a spatial resolution of 0.5 mm. However, it still lags behind the capabilities of ICA, which allows for a time resolution of 8 milliseconds and a spatial resolution of 0.2 mm [8].

It should be remembered that the dissemination of CT examinations are the most serious source of exposure to ionising radiation in the modern world. In the 20 years following 1990, the use of CT in the United States has increased approximately 20-fold. The share of radiation from diagnostic imaging sources in the total radiation exposure has increased from 20% of the total effective radiation dose (ERD) per capita to over 50%, half of which was related to CT [9].

In computed tomography, we use Roentgen rays (X-rays). They are a type of non-particle electromagnetic radiation with a wavelength of 5 pm to 10 nm, located in the electromagnetic radiation spectrum above ultraviolet, partially overlapping with gamma radiation. They are formed in X-ray tubes during the braking of the stream of accelerated electrons that are released from the cathode at the anode of the tube. Braking radiation (involving characteristic radiation, depending on the anode material) is ionising radiation, which means it can cause the release of free electrons as they pass through matter and can cause damaging effects to the tissues of the living organisms exposed to them.

The harmful effects of ionising radiation can be divided into stochastic and deterministic, or early and late, according to another division. Stochastic radiation causes effects where the likelihood (but not severity) is directly proportional to the dose. This include malignant tumours and damage to the genetic material. If they arise in the reproductive cells of the body, they can lead to genetic disorders in offspring. These effects occur after a long latency period—from five to 20 or even 40 years after the exposure to the radiation. Deterministic radiation causes effects that are dose-dependent in their likelihood and severity, but do not occur below a certain dose level (threshold dose). These include, among others, cataracts, hair loss, pulmonary fibrosis, necrosis of the digestive tract, skin erythema, skin necrosis and radiation sickness. These effects occur immediately after the exposure to the radiation, most often within 2 to 4 weeks. They are associated with an exposure to high doses above 100 mGy. With regard to the stochastic effects, the concept of a linear, non-threshold probability of their occurrence is adopted, where even the lowest dose of radiation causes a certain risk of cancer. The doses used in diagnostic imaging, referred to as low linear energy transfer (LET) radiation, are typically below 100 mSv and have stochastic effects [8,10]. It is assumed that the stochastic effects occur randomly and that the risk of their occurrence depends on the type of ionising radiation, the type of tissue irradiated and the age of the examined person. It is believed that the dose fractionation does not significantly modify the stochastic risk and that the stochastic risk is cumulative, increasing with subsequent exposures [8].

Determining the degree of risk for the doses used in diagnostic imaging is not easy. Researchers have learned about the effects of ionising radiation on the human body from catastrophic events, such as the analysis of the effects of the nuclear explosions in Hiroshima and Nagasaki and atmospheric nuclear test explosions; disasters related to damaged nuclear reactors, in particular in Chernobyl (1986) and Fukushima (2011); and errors in imaging diagnostics related to exceeding radiation doses. The source of this information is also stems from research on the occupational exposure to radiation; the exposure from medical sources, e.g., in patients with tuberculosis who received high doses of X-rays as a result of repeated fluoroscopy; the analysis of natural environmental exposures related to living in areas with high background radiation; and artificial exposures, e.g., related to the use of building materials containing radioactive elements. Based on such data analysis, risk models were constructed [8].

The most widely accepted cancer risk models are presented in the Biologic Effects of Ionizing Radiation (BEIR) Committee report. The committee is part of the American National Research Council, operating under the auspices of the US National Academy of Sciences. It publishes reports on ionising radiation effects. In the VII Report of 2006, a linear, no-threshold correlation was assumed between the exposure to radiation and the risk of cancer. According to this model, even the lowest dose of radiation carried some risk of cancer [11].

According to the BEIR VII report, for every 100,000 people who received an overdose of ionising radiation of 100 mSv, the lifetime attributable risk (LAR) was 510 in 100,000. Therefore, five out of 1000 people exposed to such radiation would die from malignant tumours caused by this radiation. For a radiation dose of 10 mSv, the lifetime LAR would be approx. one in 1000. On the other hand, the number of all the cases of malignant neoplasms, not only the fatal cases, predicted in this model was twice as high [9].

A significant proportion of the 25,000 survivors of the Hiroshima and Nagasaki nuclear explosions received radiation doses below 50 mSv. This group included individuals of all age categories and who had not been selected based on any underlying disease. However, it was an ethnically homogeneous group. The main negative effect in this group was an increase in the number of malignancies [8].

In 2007, the observations of 407,391 nuclear industry workers from 15 countries were published. The period covered by the study was as much as 20 years, which resulted in over five million person-years of observation. It involved the largest cohort to date, was conducted using accurate dosimetry and involved employees from many ethnic groups. The disadvantage of this study was that 90% of the study group was comprised of men who received as much as 98% of the cumulative dose of radiation. Additionally, it had lack of reference to the time when the cumulative dose was received by each of the participants. However, people employed for less than 1 year were excluded from the study. Most of the workers were employed in nuclear energy production facilities; the remaining plants specialised in various types of activities, including scientific research, waste management and the production of nuclear fuel, isotopes and weapons. The workers were exposed to X-rays and gamma rays. Approx. 90% of the people received cumulative doses below 50 mSv and the average dose was 19.4 mSv. Less than 0.1% received cumulative doses greater than 500 mSv. The study, therefore, provided information on the effects of the exposure to radiation doses similar to those used in cardiac CT scans. The authors reported an excessive relative risk of all-cause mortality of 0.42/Sv (0.00042/mSv) and a statistically significant increase in the excessive relative risk with the increasing radiation doses. The increased risk of all-cause mortality was mainly due to the increases in mortality from all cancers except for leukaemia and lung cancer. Among the 31 types of malignancies that were analysed, a significant relationship was identified for lung cancer and a borderline significant relationship for multiple myeloma as well as unspecified and secondary cancers. The doses received before age of 35 were associated with a lower risk for all cancers, except for leukaemia, than the doses received later [8,12].

The authors of a paper published in 2005 presented three cases of transient, bandage-shaped hair loss in the patients who underwent two DSA studies of cerebral vessels, two or more cerebral CT perfusion examinations with a tube current of 200 mA and several CT scans of the brain without CM within 15 days of subarachnoid haemorrhage. The radiation exposure to the scalp was estimated to be approximately 1.93 Gy in each cerebral CT perfusion study with a 200 mA tube current. The DLP of the cerebral CT perfusion protocol was 6.04 Gy·cm. The hair loss started on day 22 after the first CT scan. The loss was transient and lasted the longest at 92 days [13].

The harmful effects of ionising radiation are associated not only with an increased risk of developing cancer, but also with direct effects on the tissues and organs of the cardiovascular system. The importance of this problem is increasing due to the growing number of patients who have undergone radiotherapy, going on to live their lives as cured cancer patients, and due to an increasing exposure to ionising radiation associated with medical diagnostics.

Most experimental and clinical studies relating to the effects of radiation on the cardiovascular system involve doses above 2 Gy. The excess relative risk per Gy for cardiovascular disease is estimated to be in the range of 0.1–0.2 1/Gy and is lower than the cancer risk [14].

Cardiovascular disease caused by high doses of ionising radiation has been observed in American radiologists, emergency workers who entered the Chernobyl zone in 1986–1987 and were exposed to doses above 150 mSv, British Nuclear Fuels Plc employees, adults who were treated using radiotherapy in childhood and adolescence for cancer, women after radiotherapy treatment for breast cancer and in survivors of a nuclear explosion with a radiation exposure above 500 mSv [15]. A study involving a group of more than 70,000 women diagnosed with breast cancer in Denmark and Sweden between 1976 and 2006 showed an increased risk of ischaemic heart disease, pericarditis and valvular disease in the patients treated for left breast tumours who received higher radiation doses to the heart area (mean 6.3 Gy) compared to the women treated for right breast tumours (mean cardiac dose 2.7 Gy) [16,17]. The cardiovascular mortality was also significantly higher in a case-control study of Chernobyl clean-up workers between 1992 and 2006 who were exposed to an average external gamma radiation dose of 128 mSv. A possible association between increased cardiovascular mortality and low radiation doses (with mean cumulative radiation doses of 20.7, 24.9 and 21.5 mSv, respectively) was evident from studies of nuclear workers [15,17]. In an analysis of the studies on the effects of whole-body irradiation with an average cumulative dose of less than 0.5 Sv or less than 10 mSv/day, four categories of cardiovascular diseases were assessed, namely ischaemic heart disease, non-ischaemic heart disease, cerebrovascular disease and other cardiovascular diseases. The relative excess risk values were 0.1/Sv, (95% CI) for ischaemic heart disease, 0.21/Sv, (95% CI), for cerebrovascular disease and 0.19/Sv, (95% CI) for non-ischaemic cardiovascular disease and stroke [17]. A study on the association between occupational exposure to low-dose ionising radiation in 11,500 workers in diagnostic medical facilities in South Korea and the incidence of cardiovascular disease estimated the relative excess risk for all cardiovascular diseases at ERR/100 mGy to be 0.14 [17].

The primary and initial cause of cardiovascular disease induced by ionising radiation is vascular endothelial damage. The primary mechanism of endothelial damage is oxidative stress, defined as an imbalance between the formation of oxygen free radicals and the activity of enzymatic and non-enzymatic antioxidant mechanisms. Its consequences are DNA damage, changes in the gene expression (epigenetic), mitochondrial dysfunction, increased production of pro-inflammatory cytokines, activation of the mechanisms of cell ageing (senescence) and cell apoptosis.

In the first stage of the interaction between radiation and tissue, the water radiolysis products are formed, including hydroxyl radicals (-OH), hydroperoxyl radicals (HO_2_^−^) and hydrogen peroxide (H_2_O_2_). They are rapidly degraded, but the oxidative stress persists after the irradiation due to the endogenous cellular production of the reactive oxygen species mediated by the mitochondria [16].

Ionising radiation also causes reduced proliferation and apoptosis of the smooth vascular wall muscle cells [17].

In the next stage, these processes contribute to the formation and progression of atherosclerosis. The radiation-induced atherosclerotic plaques show a high inflammatory activity and have a reduced fibrous cap [14]. Following atherosclerosis, vascular stenosis develops for up to several decades after the exposure to ionising radiation. The atherosclerotic plaques can cause acute coronary syndromes, cerebrovascular incidents and peripheral ischaemia in the large and medium-sized arteries, and coronary microvascular dysfunction can lead to heart failure with a preserved ejection fraction [17].

Ionising radiation also leads to damage of the microcirculation. The radiation-induced damage of the capillary bed causes fragile and bleeding-prone telangiectasias, which develop 6 months to years after the radiation exposure. The radiation-induced damage of the renal microcirculation, or atherosclerosis of the renal artery, can lead to renin hypertension. Damage to the microcirculation in the lungs can lead to pulmonary hypertension and additional myocardial strain [14].

The radiation-induced fibrotic processes are a consequence of an inflammatory response involving the mast cells mediating the collagen deposition in the cardiac tissues. This process produces senescent fibroblasts that are metabolically active for many decades and produce increased amounts of collagen. Such reactions to radiation are observed in all tissues, including the heart. They can lead to cardiac arrhythmias [14].

After high-dose irradiation above 40 Gy, one of the earliest adverse effects is pericarditis, which appears after a few months and is characterised by the exudation of protein-rich fluid in the pericardial sac. In the subsequent course, it can lead to chronic constrictive pericarditis due to the formation of fibrous tissue, causing the thickening and rigidity of the pericardial sac. However, this is now extremely rare during radiotherapy due to the early initiation of prophylaxis [16].

Ionising radiation induces the osteogenic transformation of the valve interstitial cells and increases the production of osteogenic enzymes and cytokines and the deposition of the calcium phosphate deposits, with a consequent impairment of the valve function (insufficiency and/or stenosis) [14].

## 2. Dose Definitions and Applications in Computed Tomography

In radiological practice, we encounter the terms CTDIvol, the volumetric computed tomography dose index, and DLP, the dose-length product, which are calculated using the algorithms of the CT machine, presented on the screen of the CT scanner work console and saved in the DICOM format in the radiation dose structured report (RDSR). In 1981, the definitions of the computed tomographic dose index (CTDI) and the multiple scan average dose (MSAD) were introduced. The definition of the CTDI has changed as a new generation of CT technologies have developed. The implementation of multi-slice CT scanners and CT scanners that use wide beams of radiation has affected the CTDI’s ability to determine the radiation dose accurately. Since 2002, device manufacturers have been required to display the tomographic volumetric dose index (CTDIvol) and the dose-length product (DLP) on the operator’s console. The predicted value of the CTDIvol appears on the console after the topogram is executed and before starting the actual study. It can be used to estimate the dose even before the test is performed [1,18,19]

### 2.1. MSAD—Multiple Scan Average Dose

The MSAD determines the average radiation dose delivered during the examination to the covered area when scanning in multiple, continuous layers. It is a measure of the average absorbed dose, expressed in mGy. It is defined as the average dose in the central layer of a series of multiple layers (each of a certain thickness) when there is a constant gap between the successive layers. To measure the MSAD, one would place the radiation detector in a phantom simulating the patient (an anthropomorphic phantom) and perform a CT scan according to a specific protocol, taking the dose readings layer by layer. The MSAD value is the average dose in the centre area of the study. It is typically 1.25–1.4 times the dose of a single layer because it includes the measurements resulting from the exposure of the overlapping adjacent layers and from the diffuse radiation. This parameter did not catch on because the direct measurement of the MSAD requires multiple exposures, and in the early years of CT, testing was very time consuming. In practice, the tomographic dose index (CTDI), an alternative, more convenient way of dose estimation, is preferred, which saves a significant amount of time. In this case, the MSAD is defined as the ratio of the ply width to the ply spacing multiplied by the CTDI. If the pitch factor is one, then the MSAD = the CTDI [1,1,18].

MSAD = T/I CTDI

### 2.2. CTDI—Computed Tomography Dose Index

**Figure 1:**
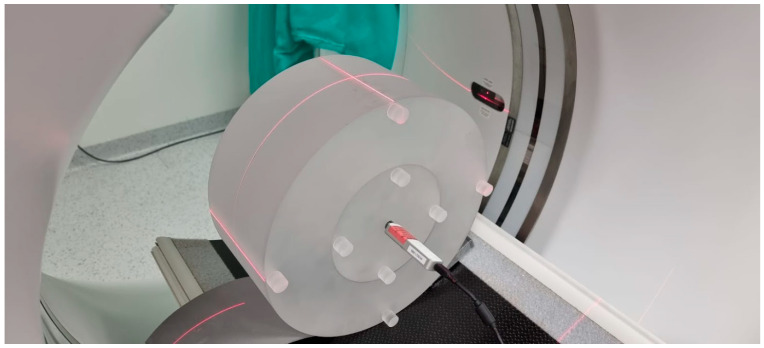
Acrylic phantom used for dosimetry in CT. Here, a 32 cm diameter phantom (torso) with nine holes. A pencil probe sits in the central hole with an ionization chamber. The visible laser lines position the phantom in the gantry.

This is the dose measured during a single rotation of the lamp detector system using a pencil ionisation chamber with a measuring part length of 10 cm. The ionisation chamber is placed in a 14 cm-long cylindrical phantom, composed of plastic (PMMA) with a diameter of 16 cm (head phantom) or 32 cm (torso phantom), as shown in Figure 1.

The measurements are taken in the holes located along the axis of the phantom and in the four holes on its perimeter, 1 cm below the surface of the phantom at every 90 degrees. It is not an anthropomorphic phantom as the measured dose is the dose absorbed in the air, not in the human body. The weighted average is calculated from the measurement in the centre of the phantom and the arithmetic mean of the measurements from the four circumferential holes. The current definition of the index was standardised by the International Electrotechnical Commission (IEC) in 2002. Its main advantage is that it can be used to estimate further indexes used in CT dosimetry, such as the volumetric tomographic dose index (CTDIvol) and the dose-length product (DLP) [1,1,12,18].

### 2.3. CTDIvol—Volumetric Computed Tomography Dose Index

The CTDI value is determined by measuring the ionisation chamber during a single revolution. In the sequential mode testing, the lower the dose, the greater the table travel between the successive layers. In the spiral (helical) mode testing, the lower the dose, the greater the spiral pitch (pitch factor) [19]. While the CTDI refers well to the sequential scanning modes, the helical (spiral, or otherwise: volumetric) mode of scanning is so different that the measurements from a dose during a single revolution are insufficient. In 2002, the concept of the volumetric computed tomography dose index (CTDIvol) was introduced, which takes into account, among other things, the pitch factor during the helical scanning. A requirement has also been introduced for the CTDIvol value to be presented on the tomograph operator’s console. This value does not have to be obtained on a specific camera. Instead, it may simply be the typical value for a specific camera model. During the specialist tests, in the case of the measurements for the spiral mode of acquisition, the measurements are taken using the sequential mode. For all the other parameters (voltage, current, collimation, etc.) the measurements are taken in the spiral mode, and the calculations are made depending on the pitch [19].

CTDI = CTDIvol if pitch = 1

The formula CDTIvol = n − CTDIw is used for the mode in which the camera performs n lamp revolutions in one table position, without moving the table.

The 2002 definition for the pitch can be stated as CTpitch factor = Δd/NT

With the advent of multi-slice computed tomography and the extension of the radiation beam—now up to 16 cm—the method of measuring the dose in specialised tests had to be modified. In the case of wide radiation beams, a 10 cm-long ionisation chamber does not register a large part or even most of the scattered radiation. Sometimes it even registers a part of the radiation beam itself, which can exceed 10 cm in width. Thus, the dose is underestimated. This is not significant in the case of beams up to 4 cm wide, while in the case of wider beams, underdosing is significant. This problem is addressed by the 2012 standard. The measurement must be taken using an ionization chamber with a length of at least 4 cm greater than the beam width. Therefore, 10 cm chambers can be used for beam widths up to 6 cm. In the case of wider beams, e.g., a 16 cm beam, longer ionisation chambers should be used, in this case 20 cm. If such a chamber is not available, then a standard chamber with a length of 10 cm can be used, but the measurements should be taken several times. The ionisation chamber should be shifted in relation to the axis of the gantry, and the measurement results should be added. The 2012 standard also defines the CTDIvol definition for the shuttle acquisition method used in the organ perfusion studies. The device console should also display information about the diameter of the phantom (16 cm or 32 cm) used for the CTDIvol measurement. The smaller the phantom, the greater the marked dose. The dose for the same exposure parameters will be approximately twice as high when using the 16 cm phantom compared to the 32 cm phantom. In the specialised testing, the deviation of the CTDIvol from the value displayed on the CT console is expected to be less than ±20%.

### 2.4. DLP—Dose-Length Product

The dose-length product (DLP) values are usually presented to the CT operator at the end of the examination and provide a convenient summary of the total amount of radiation that has been emitted to the patient. The DLP was defined as the product of the CTDIvol and the scan length (L), expressed in milligreys × centimetres. The DLP determines the radiation dose used in the examination, depending on the scope of the study, which is not taken into account by the CTDI and CDTIvol. It is important to note that this is still not the patient dose. When examining the different body regions, the DLP values should not be added together to calculate the patient dose. The DLP value obtained from each of the study ranges should be multiplied each time by a different organ conversion k-factor [1,8,19,20].

### 2.5. SSDE—Size-Specific Dose Estimates

The SSDE is expressed in milligrays (mGy) and requires the determination of the patient’s body size. The bilateral dimension (LAT) can be determined from the topogram. If two topograms are planned in the study protocol for the orthogonal projections, then the anteroposterior dimension (AP) can be determined from the second topogram. After the examination, the AP and LAT dimensions can be marked from the CT scan axial images. The dimensions of the AP and LAT are added together or the effective diameter is determined, i.e., the square root of the product of the AP and LAT. Then, from the appropriate tables, the conversion factor for the LAT dimensions or the converted AP and LAT are multiplied by the CTDIvol values from the CT device for a given examination. The coefficients are the same for all the study types but different for the different phantoms used for the dose estimation. They were created based on the Monte Carlo calculations for anthropomorphic phantoms [1,18].

### 2.6. ED—Effective Dose

The effective dose (ED) reflects the biological risk of the radiation exposure and corresponds to the dose received by the whole body. Uniform irradiation is needed to generate the same stochastic risk as the dose delivered to a part of the body during a particular CT scan. It is used to relate the different doses received by the different tissues to the total sum of the stochastic effects. It allows for the dose in a CT scan to be compared to the doses received in the other medical tests [8,21].

Its definition was introduced by the ICRP in 1991. It is calculated by adding the doses absorbed by all the organs under examination and multiplying them by the appropriate weighting factors, taking into account their sensitivity to radiation [5,8,19,21].

**Figure 2:**
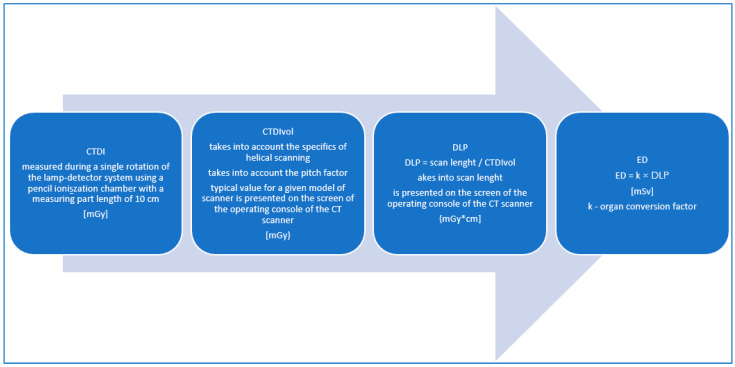
Methodology of the radiation dose estimation in the CT examinations.

The unit of measurement for the effective dose is the sievert (Sv) or millisievert (mSv) [5,8,19,21], as shown in Figure 2.

The effective dose is considered the best parameter to quantify the radiation received by the patients undergoing the ionising radiation testing and to compare the risks associated with the different types of examinations. In the CT scans, the effective dose is most commonly estimated by multiplying the dose-length product (DLP) value displayed on the dose report of each CT scan by the organ conversion k-factor.

ED = k × DLP

The methods for determining the tissue conversion factors (k) can be varied. They can be determined by dividing the effective doses, calculated using the Monte Carlo simulations performed in math or voxel phantoms, by the DLP values for the relevant studies [19,22]. Commercial CT dosimetry software packages can be used [20]. The direct radiation measurements in the anthropomorphic phantoms can also be employed by using radiochromic films (RCF) or metal-oxide-semiconductor field-effect transistor (MOSFET) radiation detectors [23].

The normalised organ conversion k-factor has different values for the different body regions and takes into account the radiosensitivity of the organs in the body region that is under examination. The adopted conversion factors are independent of sex and age, and they determine the risk for the theoretical body of a 30-year-old hermaphrodite. They are also independent of the patient size. However, in the case of a specific patient, the actual risk of cancer may be up to three times higher or lower [8,23].

The definition of the effective dose introduced by the ICRP refers to the weighting factors that take into account the sensitivity of the organs to radiation. They have already been modified and will most likely continue to be changed in the future as knowledge advances. Published in 2007 (ICRP publication 103), a new set of tissue weighting factors increased the effective dose value for cardiac imaging by approximately 30–50% compared to the previous versions from 1996. This was mainly due to an increase in the tissue weighting factor for breast tissue, the value of which increased from 0.05 to 0.12. Previously, it was 0.15 in 1977, but was reduced to 0.05 in 1991. Since the breasts are directly irradiated by the X-ray beam during CT scans of the heart, together with the lung dose, they are the main dose-determining organs that are effective in cardiac CT scans. In cardiac CT scans, the equivalent organ dose (expressed in mSv) to the breast may be significantly higher than the effective dose (also expressed in mSv). Therefore, confusion between these terms should be avoided [8,19,21,23].

Due to its imperfections, the effective dose cannot be used to assess the radiation risk for a particular patient. The ICRP emphasises that the effective dose is intended for use in radiation protection and should not be used for the epidemiological assessment or the estimation of specific human exposures. Nevertheless, it remains the most widely used parameter to compare radiation exposure across the test methods and protocols [19,21,23].

## 3. Radiation Doses in Cardiovascular CT Scans—A Review of Reports

The radiation doses used in cardiovascular CT scans are not low. During these examinations, the largest doses of radiation are absorbed by the lungs and breasts, which are the organs most sensitive to radiation-related damage. Younger patients are clearly more sensitive to radiation and are more likely to develop lymphoma and breast cancer, so attention should be paid to the radiation doses used during cardiac CT scans in children and young adults. Women who are screened during breastfeeding are particularly at risk of developing breast cancer. The radiation doses to which the breasts are exposed in cardiac CT examinations exceed the doses associated with mammography in two projections (3 mGy). In turn, the risk of lung cancer is potentially higher in the elderly due to advanced age and the possible synergistic carcinogenic effects of tobacco smoke and ionising radiation [8,10].

In the CORE-64 study, the mean effective dose on the coronary CT scans was 19 mSv (range 16–26 mSv), as determined by the Monte Carlo simulations for the 64-slice Aquillion scanner and calculated according to the ICRP report 103 of 2007. The mean effective dose for the entire cardiac CT protocol, including the topograms, calcium score, contrast bolus monitoring and coronary vessels CT was 22 mSv (range 18–30 mSv). The calcium score doses ranged from 1.7 to 2.6 mSv. The contribution of the topograms and contrast agent bolus monitoring was very small, at ≤0.3 mSv. The highest mean organ equivalent doses associated with the cardiac CT scans for the normal body patient models were observed as 38 mSv for the breasts, 35 mSv for the lungs, 32 mSv for the liver, 29 mSv for the stomach and 27 mSv for the oesophagus. In the case of distant organs, the dose was 0.2 mSv or less for the bladder, ovaries—0.2 mSv or less for the ovaries and 0.02 mSv or less for the testicles [24].

For the study, the doses of over 35,000 coronary artery CT scans that were performed at the University Center in Nanjing (China) in 2007–2016 with the use of 1st- and 2nd-generation dual-source scanners (Somatom Definition and Somatom Flash, Siemens Healthineers, Forcheim, Germany) were analysed. I median values were 47.4 mGy for the CTFIvol (value range 25.1–61.1 mGy), 661.2 mGy*cm for the DLP (value range 315.2–940.0 mGy*cm) and 66.9 mGy for the SSDE (value range 38.5–97.3 mGy). The radiation doses decreased after the implementation of the 2nd-generation dual-source computed tomography (Definition Flash) and reached the lowest level in the last year of the analysed period, where the CTDIvol was 23.1 mGy (value range 15.7–32.4 mGy), the DLP was 268.2 mGy*cm (value range 183.0–393.6 mGy*cm) and the SSDE was 32.5 mGy (value range 22.9–45.2 mGy). The median CTDIvol for the studies in the retrospective acquisition protocol was 57.9 mGy (range 47.2–59.0 mGy), approximately 116.9% higher compared to the prospective sequential acquisition and 185.2% higher compared to the prospective helical with the use of a high pitch. After the CABG, the patients received higher radiation doses than a classic coronary CT scan, while the highest doses were received by the patients with arrhythmia. The calcium score studies were excluded from the dose analysis in this study [25].

In the analysis of 5000 patients examined in German centres from the years 2009–2014, the median DLP in the calcium score scan was 40 mGy*cm. In the case of the CCTA, it was 255 mGy*cm, which decreased from 397 mGy*cm at the turn of 2009 and 2010 to 176 mGy*cm from 2011–2017. In this study, a significant percentage of coronary CT examinations in the prospective acquisition protocol was noteworthy, which increased from 67.5% to 82.2% in the analysed period, while the share of the studies with retrospective acquisition without the dose modulation decreased from 5.3% to 1.6% [26].

In a paper from 2012, A. Sarma et al. provided the estimated radiation doses for the calcium score scans at an average of 3.0 mSv (dose range 1.0–12.0 mSv), for coronary CT at an average of 8.7–16 mSv (dose range 1.0–32.0 mSv) and for the triple rule-out study at an average of 4.0–31.8 mSv (range 7.5–19.4 mSv). These doses, converted to the DLP using the value of the coefficient k = 0.017 were 214 mGy*cm for the calcium score, 621–1143 mGy*cm for the coronary arteries CT and 286–1386 mGy*cm for the triple rule-out study [10].

When comparing the doses used in the studies pooled from the four centres in Saudi Arabia, the median DLP values were 320–432 mGy*cm on the Siemens Somatom scanners and 1112 mGy*cm on the Philips Ingenuity scanner (Philips Healthcare, Amsterdam, The Netherlands). The authors mentioned that the exposure parameters, such as the tube voltage (kVp), exposure time product (mAs), pitch settings, layer thickness and scan length, may have affected the patient radiation exposure and image quality. One of the labs (equipped with the Somatom scanner), regardless of the patient’s BMI and clinical conditions, used a constant tube voltage, rotation time, pitch and layer thickness, which overexposed the patient to the radiation dose. The high dose at the Philips facility was not associated with high DLPvol value (33.1 mGy) but with the use of long scan ranges. The mean effective dose on a coronary CT scan was 15.2 ± 8 mSv and ranged from 1.2 to 61.8 mSv. The highest DLP value in the analysed studies was 3277 mGy*cm. Since the average expected radiation risk from the coronary CT scans was one in 1000, the risk from this highest dose could be estimated as one case of malignancy in 300 scans. This increased cancer risk was due to the non-optimised irradiation procedures and was immediately corrected [27].

In the CRESCENT study, conducted from April 2011 to July 2013, the coronary artery calcium score was performed on a group of 242 patients with stable angina pectoris referred to the outpatient clinics of four Dutch hospitals. This was followed by the CCTA if the calcium score did not exceed 400. In this study, 117 patients were included. The mean radiation dose from the calcium score was 2.4 mSv and 8.5 mSv from the full cardiac CT scan [28].

In the 2017 PROTECTION VI study (the prospective multicentre study on radiation dose estimates of cardiac CT angiography), the median total DLP of all 4502 patients was 252 mGy*cm (IQR 154–412 mGy*cm). This was a prospective, worldwide, multicentre, observational, survey-based study to assess the radiation exposure during the cardiac CT examinations in everyday practice. It involved 4502 patients from around 70 centres in Europe, North America, South America, Asia and Australia. It did not benefit from financing from its equipment suppliers. A total of 435 radiologists and cardiologists from 62 different countries were invited to participate in the study. The DLP values for the coronary CT alone were 195 mGy*cm, corresponding to an effective dose of 2.7 mSv using the conversion k-factor for the chest CT scans (0.014 mSv/mGy*cm) or an effective dose of 5.1 mSv using the conversion k-factor recently postulated for the cardiac CT scans (0.026 mSv/mGy*cm). In 2017, compared to the 2007 study, a significant 78% reduction in the DLP was observed (*p* < 0.001) [29]. The difference in the median dose from the individual centres was as much as 37-fold (from 57 to 2090 mGy*cm). The reduction in the radiation dose did not increase the rate of the non-diagnostic coronary artery CT scans (1.9%). It should be noted that 78% of the studies were performed using prospective, sequential acquisition protocols. [29,30,31] The median radiation dose for the studies using a conventional tube voltage (120 kVp) was estimated to be 4.3 or 8.1 mSv, using the thoracic or the recently published cardiac DLP to effective dose conversion factor of 0.014 or 0.026 mSv/mGy*cm, respectively [29].

A summary of the dose values from the publications in question is shown in Table 1.

In the conclusion of the PROTECTION VI study, a diagnostic reference level for the coronary CT scans was proposed. This value was usually set at the 75th percentile of the dose for a typical patient size and for a particular radiological procedure. It was not a recommended or preferred dose, but rather a value to aim for. The authors suggested that a new DLP diagnostic reference level of 400 mGy*cm should be considered [29]. As a reminder, in the PROTECTION I study conducted 10 years earlier, the DLP reference level for the angio-CT of the coronary vessels was set at 1200 mGy*cm (17 mSv) [19]. The achievable dose, on the other hand, was the level set at the 50th percentile [25].

The reference levels of the radiation doses for the coronary CT under the Ionising Radiation (Medical Exposure) Regulations in the UK were 380 mGy*cm for the retrospective acquisitions with ECG monitoring and 170 mGy*cm for the prospective acquisitions without padding [32].

Based on a survey conducted in 2016/2017 under the ICRP rules in 55 centres on 338 patients in Australia, the national dose reference levels were established for the CCTA with a DLP of 268 mGy*cm and for the CS with a DLP of 137 mGy*cm [33].

A similar study was conducted in France in 2013 in eight hospitals. A total of 460 CCTA studies were analysed. The reference dose levels were proposed for the retrospective studies with an ECG-gating DLP of 870 mGy*cm and a DLP of 393 mGy*cm for the prospective studies with an ECG-gating DLP of 370 mGy*cm [34].

In 2016, a similar study was carried out in 11 centres in Saudi Arabia, analysing information from the RIS and PACS data of the studies of 197 patients. The studies in the prospective protocol were used in 55% of the patients. The reference dose levels were set at a DLP of 395 mGy*cm for the studies in the prospective protocol and at 1057 mGy*cm for studies in the retrospective protocol [35].

The more recent data came from a single-centre analysis in Taiwan involving 445 patients that was studied from February 2017 to December 2019. The following reference levels were established—for the CCTA, a DLP of 560.1 mGy*cm and for the calcium score, a DLP of 39.2 mGy*cm [36].

The dose reference values (DRLs) are shown in Table 2.

Einstein et al. estimated the lifetime risk of cancer in a 20-year-old woman as 0.7%, following a single coronary CT scan in a protocol without applying a dose reduction. Hurwitz et al., in turn, estimated that the excess relative risk in a 25-year-old woman who underwent a coronary CT scan ranged from 1.4% to 2.6% for breast cancer and from 2.4% to 3.8% for lung cancer [10,37,38]. In the whole coronary CT cohort, the estimated mean lifetime risk of radiation-induced cancer was 0.13% [10,39]. In comparison, the relative risk of lung cancer associated with an effective dose of 1000 mSv (approximately 50–100 coronary CT scans) was 2.9. They can be compared to the relative risk of lung cancer in tobacco smokers, which—according to the statistics of the US National Cancer Institute—was 4.9 in people who smoked up to 15 cigarettes per day and 13.3 in people who smoked up to 25 cigarettes per day [21].

It should be noted that multiple, repeated CT scans can lead to high cumulative ERDs (50–200 mSv). Compared to the dose from a single CT scan, a cumulative dose of 120 mSv (equivalent to 12 coronary angio-CT scans) can increase the lifetime cancer risk from 1/1000 to 1/82 [9].

The effect of the length of the imaging interval on the risk of developing malignancies remains unclear, partly because of the undefined effect of the cellular repair mechanisms. However, it is assumed that the radiation risk of two CT scans is approximately twice that of a single scan, regardless of the time interval between them [10].

The median age in the PROTECTION VI study was 60 years. An effective dose of 5 mSv at this age increased the additional lifetime risk of malignancy only marginally, and the benefits of the coronary CT information significantly outweighed this risk. The estimated risk of lethal malignancy due to CT with a dose of 10 mSv was 0.05%. In contrast, the benefits of coronary CT were an estimated 50% reduction in the number of fatal and non-fatal myocardial injuries observed in the 3-year period following a CT scan [29].

In comparison, the median effective dose for a frequently performed SPECT study was approx. 10 mSv worldwide, 7 mSv in conventional chest CT scans, 14 mSv in 18F PET cardiac scans, 10 mSv in cardiac stress tests with 99mTc sestamibi, 40 mSv in thallium stress tests 210 Tl and 7 mSv in diagnostic coronary angiography [20,29,40].

The radiation doses from medical sources are often referred to as ‘background radiation’. Over one year, patients receive slightly less than half the dose associated with a routine chest CT scan (3 mSv) from background sources, including cosmic rays and radon gas. Compared to a chest X-ray in two projections, the radiation dose in a chest CT is 100 to 400 times higher [10]. The average dose level from background radiation in Poland is 2.5 mSv per year [41].

The comparison of the CT protocols should be conducted using the DLP values as all the CT systems share the same dosimetry system. Comparing the equipment or test protocols on the basis of the effective dose expressed in millisieverts (mSv) always requires the use of the same conversion k-factor. Different publications use different values, from 0.014–0.017 for chest examinations to 0.024 to 0.030 mSv/mGy*cm for postulated cardiac examinations [24,27].

Assuming there is a linear, no-threshold concept of ionising radiation harm, the estimated lifetime risk of death from cancer associated with the radiation dose received during a typical coronary CT scan (ok. 10 mSv) is 0.05%. On the other hand, ICA performed for diagnostic purposes only has a “serious” complication rate of 1.7% (including mortality: 0.11%; myocardial infarction: 0.05%; stroke: 0.07%; haemodynamic complications: 0.26% and serious contrast agent reaction: 0.37%). This should be added to the theoretical risk of future malignancy associated with the use of fluoroscopy during ICA, which is 0.02% [19].

In 2018, an expert consensus from the Society of Cardiovascular Computed Tomography (SSCT) was published referring to coronary artery examinations in women. The authors indicated that exposure to ionising radiation was a major safety concern for CT in women, and this was especially true for breast exposure. The paper stated that after a CT scan of the heart of a 60-year-old woman, her LAR rate of malignancy was 0.22% (one in 466). In the estimation of this risk, a higher number of effective doses resulting from the study were assumed than are currently commonly used. In the studies on an anthropomorphic phantom performed using a 64-slice apparatus in a retrospective spiral protocol with ECG monitoring but without dose modulation, the absorbed dose in the breast was 82.9 mGy. On the same phantom, using dose modulation on a 320-slice device, the dose was reduced by 79%—to 17.5 mGy. This showed how modern dose reduction techniques can reduce radiation exposure. With a skilful use of the CT study protocols, the dose is more of a concern for certain studies, such as myocardial perfusion imaging using SPECT, PET and ICA [40].

The authors of the study do not recommend the use of breast shields. However, in the process of positioning the patient, they recommend the manual movement of the movable part of the breast outside the field of CT imaging of the heart. This makes it possible to reduce the dose absorbed by the breasts, reduce the absorption of the radiation during acquisition and allows for the use of a lower CT tube current. This reduces the effective radiation dose to 33% compared to men with similar BMI values [40].

The discussed publication also raised the important problem of CT examinations during pregnancy. The authors confirmed that examinations that use ionising radiation during pregnancy may be performed only when the expected results may change the medical management. The radiation risk to the foetus was mainly due to the diffuse radiation from within the imaged part of the patient’s body. The radiation doses to the foetus during chest CT or CT lung angiography to exclude pulmonary embolism were low. In the case of testing to exclude pulmonary embolism, it was estimated to be within 0.02 mGy, so the risk of non-stochastic foetal damage in the case of CT limited to the chest was negligible. Higher doses were associated with the studies that directly involve the foetus. The foetal dose for the CT angiography of the chest, abdomen and pelvis was 13 mGy. The authors of the guidelines referred to the opinion of the American College of Obstetrics and Gynecology (ACOG), which states that, if clinically indicated, angio-CT should not be withheld in pregnant patients. Instead, before the test is performed, the risks and benefits should be considered and discussed. The assessment of stochastic risk to the foetus is very difficult. The authors of the study referred to the models that allowed for estimating the excess risk of malignant neoplasm associated with a radiation dose of 10 mGy on the foetus from one in 4545 in the high-risk model to one in 1667 in the low-risk model. It should be noted that, although the iodinated contrast agents may cross the placenta into the foetal circulation during pregnancy and may be detectable in the amniotic fluid, there is no evidence of teratogenic, mutagenic or other foetal harm [40].

## 4. Optimization Methods—Reduction in the Radiation Dose

In each study, the capabilities of the equipment and the ability to use the protocols should be adapted to the clinical problem and the patient characteristics in accordance with the ALARA (as low as reasonably achievable) principle. The opportunities to optimise the radiation dose may vary, depending on the conditions of the examination and the type of equipment.

The optimisation methods and reductions in the radiation dose in the CT examinations include the following.

- CT tube voltage reduction
- ECG-monitored radiation modulation (tube current)
- Iterative image reconstruction
- Deep learning-based image reconstruction and deep learning-based image denoising
- Reduction in the scan range (scan length)
- Prospective study protocols
- Modulation of the current intensity depends on the attenuation of the radiation
- Heart rate control
- Rational use of the calcium score coronary artery calcification test
- Multi-slice, dual-source and wide-field tomography

### 4.1. CT Tube Voltage Reduction

A lot of information regarding the possibility of adjusting the tube voltage was provided by the PROTECTION studies. They were prospective, multicentre, survey studies based on the analysis of the radiation doses in CT scans of coronary arteries. Lowering the tube voltage is extremely effective in lowering the radiation dose due to its exponential reduction.

In the PROTECTION I study, the results of which were published in 2009, the radiation doses and image quality from the cardiac CT scans were compared in a group of 82 patients using a 100 kVp voltage and 239 patients using a 120 kVp voltage. At that time, out of 50 centres, only eight used a reduced tube voltage. The effective dose of radiation was estimated on the basis of the DLP values. The quality of the study was assessed by an experienced researcher on a four-point scale. The authors of the study found that the application of the voltage of 100 kVp was associated with a reduction in the median radiation dose by 53% compared to the tests with the voltage of 120 kVp. Although the image noise increased by 26.3% in the 100 kVp tests, the signal-to-noise ratio (SNR) increased by 7.9% and the contrast-to-noise ratio (CNR) increased by 10.8% due to the reduced tube voltage increased the density of the contrasted vascular lumen. Reducing the tube voltage did not impair the diagnostic quality of the image. The median estimated radiation dose was reduced from 14 mSv for 120 kVp to 6 mSv for 100 kVp [42].

In 2019, the results of the PROTECTION VI study were published, which analysed, among other things, the impact of very low (80 kVp), low (90–100 kVp), conventional (110–120 kVp) and high (130 kVp) voltages on the radiation dose in the CT scans coronary arteries. A total of 61 international research centres from 32 countries provided the imaging data and protocols for the CCTA studies performed during the 1 month period between March and December 2017. Approx. 91% of the examinations were performed using cameras with at least 128 slices [29]. Nearly 10 years after the PROTECTION I trial, the low tube voltage was used in 56% of the trials (80 kVp in 9%; 90 to 100 kVp in 47%) [29,30]. The low tube voltage protocols, 90 to 100 kVp, were less frequently used in the GE equipment (42% CCTA) compared to the others (Toshiba: 45%, Philips: 49%, Siemens: 50%). The frequency of use of the 80 kVp ultra-low potential tube was significantly higher in the Siemens scanners (17% CCTA) compared to all the other suppliers (GE: 1%, Philips: 3%, Toshiba: 4%) [30]. The radiation doses were read for each study from the DLP reports [29]. The use of the low tube voltage protocols significantly reduced the median CTDIvol to 11.1 mGy when using 90 to 100 kVp and 6.9 mGy when using 80 kVp. The application of the voltage of 80 kVp resulted in a reduction in the average DLP by 68% compared to the tests with the voltage of 120 kVp, while at the voltage of 90 to 100 kVp, the reduction in the DLP was 50% on average [19,30]. It should be mentioned that the CT examinations conducted in the process of the qualification for the TAVI procedures were excluded from the analysis due to the heterogeneity of the acquisition protocols [29,30].

The authors of the analysis of the PROTECTION VI study concluded that, taking the BMI criteria into account (80 kVp for a BMI < 25 and 90–100 kVp for a BMI 25–30), 58% of the patients tested using the conventional tube voltage were able to use the protocols with a low tube potential (90 to 100 kVp), and 44% of the patients tested at 90–100 kVp would qualify for the protocols with a very low tube potential (80 kV). The PROTECTION VI trial data showed that the dose reduction strategy of lowering the tube voltage was still underutilised in daily practice. The four centres participating in this study carried out the tests using the 120 kVp voltage only. The women and patients with a lower cardiovascular risk and lower BMI values were more often referred for the studies using the lower-than-conventional tube voltages. A strict implementation of the criteria of a BMI < 25 kg/m^2^ in the qualification for the 80 kVp test and a BMI between 25 and 30 kg/m^2^ in the qualification for the 90 to 100 kVp test would reduce the median DLP in the PROTECTION VI population by an additional 23% (up to 150 mGy*cm) [30].

In the PROTECTION VI study, lowering the tube voltage also reduced the contrast medium volume by 25% for 80 kVp (to 55–79 mL) and 13% for 90–100 kVp (to 60–80 mL) [29,30]. Some of the data indicated that reducing the volume of the iodinated contrast medium not only helped protect kidney function, but also reduced the potential effects of irradiation. In a study conducted on 245 patients in whom lymphocytes were collected before and after the chest CT scans and evaluated using fluorescence microscopy, it was shown that the patients who underwent CT with an iodinated contrast agent had a 107% increase in the amount of radiation damage to their DNA compared to the study group that was not administered a contrast agent [43].

In turn, Huda et al. performed simulations using the IMPACT program, which showed that reducing the X-ray tube voltage from 140 to 80 kV while maintaining constant mAs values will reduce the radiation dose five times [20].

### 4.2. ECG-Monitored Radiation Modulation (Tube Current)

In cardiac CT, the time of the data acquisition during the diastole, when the movement of the heart and coronary vessels is minimal, is most desirable. The earliest acquisition protocols using ECG monitoring in the cardiac studies generated a constant radiation intensity throughout the cardiac cycle. However, the modification of the radiation dose has been used for many years so that it is maximal in the diastole and minimal (approx. 20% of the maximum value) during the contraction, achieved by changing the CT tube current. This procedure can reduce the radiation dose by up to 50% without a significant loss in the image quality [19]. In the radiation modulation protocols, there is a risk of an incorrect determination of the optimal timing for the acquisition in patients with sinus tachycardia, arrhythmias or ectopic beats. The use of the maximum and minimum tube currents at the inappropriate phases of the heart cycle may cause difficulties in interpreting the images or make diagnostic examination completely impossible. Since the concept of ECG-monitored radiation modulation works most effectively in the patients with a regular, slow heart rate, a pharmacological slowing of the heart rate is recommended [19]. The radiation modulation can be omitted in the studies where cine imaging is necessary, e.g., when the valve movement or heart muscle function must be assessed. The time resolution of the apparatus should also be taken into account each time. Although there are constructions of CT machines that ensure a good examination quality even at a beat rate of 80 bpm and higher, most centres do not yet have such scanners.

### 4.3. Iterative Image Reconstructions

Until the beginning of the 21st century, CT images were obtained using filtered back projection (FBP) algorithms. The main advantages of this algorithm were the low computational power requirements and the speed of reconstruction, while the disadvantages were the rather significant image noise, especially when using low tube current intensities, poor contrast resolution and streak artefacts. Increasing the signal-to-noise ratio (SNR) required an increase in the radiation dose. The new iterative reconstruction algorithms, used in nuclear medicine since the 1980s, then came to the rescue. The advances in computational technology made it possible to implement them in CT image reconstruction.

The term ‘iterative’ (from Latin “*iteratio*” meaning repetition) means repeating the same operation in a loop until a certain condition is met. Iterative reconstruction algorithms perform calculations in a loop in a way that allows for a significant noise reduction, preserving the information about the edges and the contrast of the anatomical structures [44]. In these calculations, the data with a low statistical uncertainty are assigned a higher weight than the data with a higher statistical uncertainty. This is overlaid with the algorithms for modelling the photon interaction between the X-ray tube, the isocentre and the detector. The first iterative reconstruction algorithm used in cardiac CT was ASiR (GE Healthcare, Chicago, IL, USA), implemented in 2008. In the same year, Siemens Healthcare (Forchheim, Germany) introduced the IRIS algorithm, which was later replaced in 2010 by the SAFIRE (sinogram affirmed iterative reconstruction) algorithm, which works in both the raw data and image domains [45].

In the iterative reconstruction methods, the noise does not depend as strongly on the CT scanner tube voltage as in the FBP methods. Therefore, the IR algorithms allowed for the use of strategies to reduce the radiation dose by lowering the tube voltage. In addition, they allowed for coronary artery CT to be performed in patients with high BMI values, who previously could not be examined with high image quality due to the significant noise, and in patients with a high calcium score and implanted stents, who previously could not be examined effectively using CT due to the blooming artefacts [45].

One study showed a 24% dose reduction in the coronary artery CT with ASiR (adaptive statistical iterative reconstruction, GE Healthcare, USA) compared to the FBP, with no difference in the signal-to-noise ratio (SNR) or the contrast-to-noise ratio (CNR), but with a comparable diagnostic quality [46].

The use of the IRIS algorithm (Siemens Healthcare, Erlangen, Germany) in the cardiac CT examinations allowed for the use of a tube voltage of 80/100 kVp. In these studies, the average ED was 3.7 mSv. This was compared to the average ED of 9.7 mSv in the studies reconstructed using the FBP with the same diagnostic quality and a 120 kVp tube voltage. This represented a reduction in the radiation dose of 62% [47].

The ADMIRE (advanced model iterative reconstruction, Siemens Healthcare, Germany) algorithm uses advanced modelling to improve the image resolution and edge detection, as well as the noise reduction. This algorithm enables coronary artery CT to be performed with a low radiation dose of 0.3 mSv [45].

The PROTECTION VI study showed that the combination of the iterative image reconstruction and the reduced X-ray tube voltage resulted in a reduction in the radiation dose without compromising the quality of the examination images. The use of the iterative image reconstruction resulted in a 33% reduction in the radiation dose compared to the filtered back projection [29,30,31]

### 4.4. Deep Learning-Based Image Reconstruction and Deep Learning-Based Image Denoising

The use of artificial intelligence algorithms in image reconstruction and denoising in CT scans has increased significantly in recent years. These algorithms make it possible to obtain diagnostic quality CT images at lower doses than the iterative reconstruction used to date. It has become possible to perform CT examinations using radiation doses that previously did not allow for sufficient diagnostic quality images.

A paper comparing the images of the CT scans of the thorax, abdomen and pelvis at sub-millisievert doses that were reconstructed using iterative reconstruction and deep learning algorithms was published in 2019. The patients for the standard-dose CT examinations were additionally acquired using the low-dose examination protocols (100 kVp, 120 kVp; 30–50 mA)). The reconstructions of the images from the standard acquisitions were developed using the iterative algorithms (adaptive iterative dose reduction [AIDR] 3D, Canon Medical Systems, Ōtawara, Tochigi, Japan), while the reconstructions of the low-dose CT images were created using the iterative algorithms and deep learning artificial intelligence (Advanced Intelligent Clear-IQ Engine [AiCE], Canon Medical Systems). The mean DLP values were 567 ± 249 mGy*cm for the standard CT studies and were significantly lower for the LDCT: 49 ± 13 mGy*cm. In an image comparison conducted in an independent, randomised and blinded trial, the low-dose CT scans of the thorax were found to be 95–100% diagnostic. The quality of the images that were reconstructed using the artificial intelligence algorithms allowed for significant reductions in the radiation doses for the CT scans [48].

As the radiation dose decreased, the noise increased, which manifested itself by blurring the contours of the anatomical structures and producing low-contrast images in which the pathological changes may remain undetected. Over the past few decades, various denoising algorithms have been proposed. They can be divided into three categories: filtering in the sinogram domain (raw data), iterative reconstruction and processing in the image domain. The DLR algorithms enable the image noise reduction, high resolution and lesion recognition [49].

The DLR algorithms are the image domain restoration methods. The machine learning-based methods are aimed at automatically learning and improving the applications through experience, rather than using user-defined programmes. They are effective in denoising, which shows a non-uniform distribution in the CT images. The dynamic development of the hardware and computing techniques has led to the popularity for artificial intelligence algorithms in denoising based on convolutional neural networks. The network achieves this effect by learning from the training data based on the examinations taken at a conventional radiation dose, then relating this information to the images taken at low doses [49]. Reducing the noise without losing the important image features, such as the edges, angles and other sharp structures, is a difficult task. Kaur et al. reviewed and compared the various noise reduction techniques in abdominal and pelvic CT images [50].

**Figure 3:**
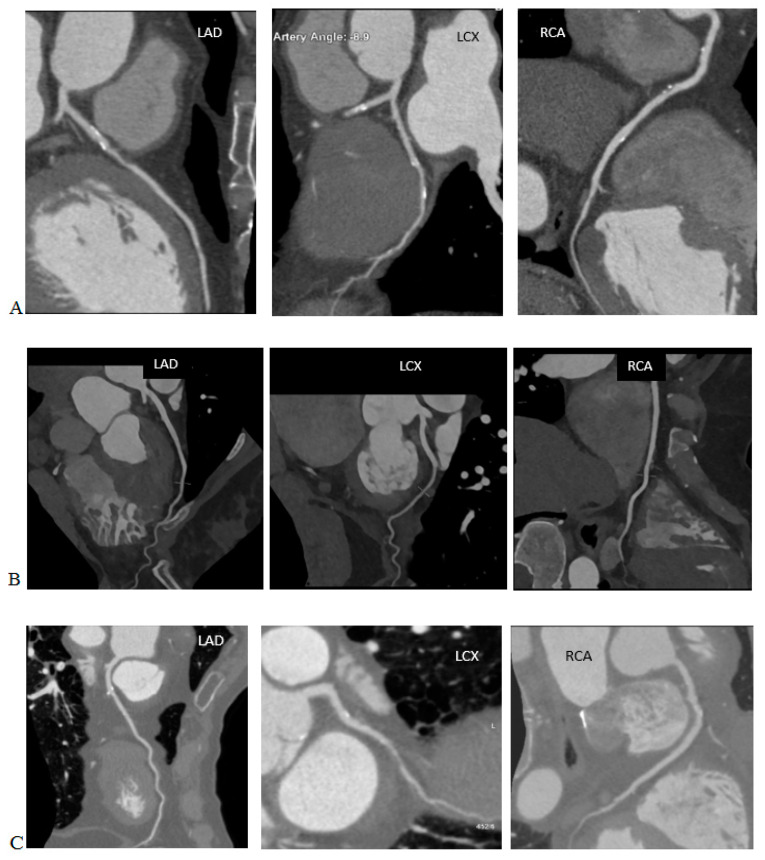

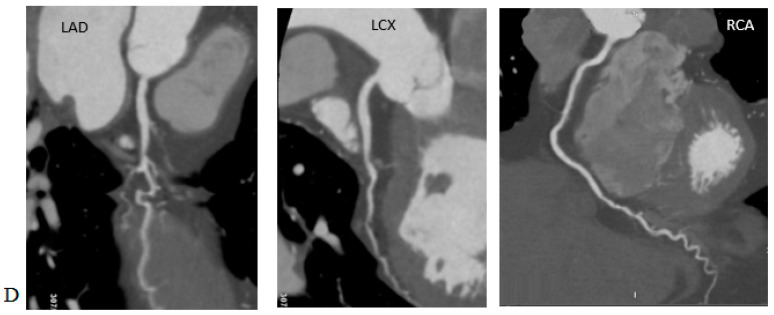
Coronary artery computed tomography scans performed on various scanners, using iterative reconstructions and AI algorithms. Note the DLP and effective dose (ED) of the studies and compare them to the image quality. (**A**) 120 kVp, DLP 1684.6 mGy*cm, ED = 23.58 mSv (k = 0.014 mSv/mGy*cm), iterative reconstruction. (**B**) 100 kVp, DLP 712.2 mGy*cm, ED = 9.97 mSv (k = 0.014 mSv/mGy·cm, reconstruction with AI algorithms. (**C**) 100 kVp, DLP 715.7 mGy*cm, ED = 10.02 mSv (k = 0.014 mSv/mGy*cm), iterative reconstruction. (**D**) 100 kVp, DLP 99.7 mGy*cm, ED = 1.40 mSv (k = 0.014 mSv/mGy*cm), reconstruction with AI algorithms.

Artificial intelligence algorithms have a high efficiency for denoising images. They also provide a better spatial resolution than the iterative algorithms, while maintaining similar radiation dose levels. Phantom and clinical studies have shown that deep learning reconstructions allow for a dose reduction of 30–80% compared to the current iterative reconstruction algorithms while maintaining a diagnostic image quality. In CT angiography, the artificial intelligence algorithms allow for fine vessel reconstructions with the examinations performed at radiation doses that would not be possible with the iterative reconstructions [51], as shown in Figure 3.

Currently, CT device vendors provide commercial algorithms for image reconstruction or image denoising based on artificial intelligence, such as the Advanced Intelligent Clear-IQ Engine (AiCE) (Canon Medical Systems) and TrueFidelity (GE Healthcare) [51].

The authors of the study, which evaluated the images of the studies of 50 patients who underwent coronary artery CT using standard- and low-dose radiation, showed that the DLR enabled a 43% reduction in the radiation dose in the CCTA with no significant effect on the image noise, stenosis severity, plaque composition or quantitative plaque volume [52].

The use of the DLR algorithm for cardiac CT in a thrombus assessment (in comprehensive stroke diagnosis, in prospective acquisition) reduced the radiation dose by approx. 40% and improved the image quality by approx. 50% compared to the IR algorithm. The mean DLP for the DLR algorithms was 106.4 ± 50.0 mGy*cm compared to the IR (176.1 ± 37.1 mGy*cm). The ED was lower for the DLR and was 1.5 ± 0.7 mSv (for IR 2.5 ± 0.5 mSv). Compared to the IR, an increase in the SNR and the CNR of approximately 51% and 49%, respectively, was shown for the DLR [53].

Kang et al. analysed the cardiac CT images of 50 patients with mitral valve prolapse and 50 patients with coronary artery disease taken using a Somatom Definition Flash scanner, (Siemens Healthineers, Germany). A neural network was involved and subjected to deep learning to reduce the noise. Two networks were subjected to training between two different domains (a low dose and a routine dose). The network designed by the authors did not require exactly matched images of the low and routine doses. It was designed to identify the distributions of the high-dose cardiac phase images and to prevent the generation of artificial features that were not present in the input images. The network performed well in reducing the noise in the input low-dose CT images while retaining the texture and edge information [54].

### 4.5. Reduction in the Scan Range (Scan Length)

In most cardiac CT scans, the length of the scanning range is 12 to 13 cm for adults, typically extending from the tracheal bifurcation to the diaphragm. The patient dose per procedure (DLP) was found to increase by approximately 5% for every one cm increase in the scan length [8,27]. Applying a “safety margin” above and below the heart for fear of missing important heart structures is not appropriate [19]. However, the scope of the examination should always be extended in the case of a vascular graft examination, simultaneous assessment of the aorta and in studies where there are other relevant clinical indications.

### 4.6. Prospective Study Protocols

In this acquisition mode, the CT X-ray tube is turned on for only a short period—in the middle of the diastolic phase—after which it turns off. During this time, the table with the patient moves to the next position, where a short acquisition in the diastolic phase occurs again. The method was named ‘step-and-shoot’. Limiting the radiation of the CT tube to only a fragment of the diastolic phase of the heart cycle allows for reducing the radiation dose by as much as 78% to 1–5 mSv [19]. The acquisition of the prospective protocol requires a sufficiently long time window for the collection of the image data, in which the movement of the coronary vessels is minimal. Therefore, it cannot be used for a heart rate that is too fast. The optimal moment of acquisition is assumed to be half of the diastolic phase—from 60% to 70% of the R-R cycle—at a heart rate no higher than 60 bpm. Accelerating the heart rate reduces the available acquisition time. Arrhythmia and ectopic beats may result in a non-diagnostic image quality [8,19]. Due to the acquisition of the data from only a small part of the R-R cycle, the prospective study protocols cannot be used where it is necessary to assess the function of the heart valves, myocardium, or the reconstruction of other anatomical structures in motion. In the analysis of the examinations performed using dual-source devices at the University Center in Nanjing (China) in the years 2007–2016, in the first year of the analysis, all the examinations were performed using retrospective protocols with a radiation dose modulation. However, in the last analysed year, approx. 2/5 studies were conducted using the prospective protocols. The median DLP value in these studies was 311.7 mGy*cm [25]. In PROTECION VI, 78% of prospective protocols were used [30].

### 4.7. The Modulation of the Current Intensity Depends on the Attenuation of the Radiation

The modulation of the current intensity takes into account the fact that the cross-section of the body is usually oval, so in the anterior–posterior dimension the radiation beam has to overcome a smaller layer of tissue compared to the bilateral dimension. In addition, the thickness of the tissue layer also changes with the movement of the table. Equipment manufacturers use software that adjusts the CT tube current intensity to the thickness of the tissue layers. In the AP dimension, the tube current is lowered, while in the double-sided dimension, it is increased. This technique is called the automatic exposure control (AEC) and can provide a significant reduction in the radiation dose with minimal deterioration in the image quality [27].

### 4.8. Heart Rate Control

In a large proportion of coronary angio-CT studies, it was necessary to reduce the heart rate to no more than 60 bpm to match the time resolution of the equipment. An increase in the heart rate of 10 beats per minute and an absence of the sinus rhythm were associated with an 8% and 21% increase in the radiation dose, respectively [29]. However, some test protocols used in newer CT scanners, and the radiation dose was lower at the higher heart rates due to a less irradiated layering between the successive rotations of the gantry [19]. The median DLP in the study of the patients with arrhythmias at the Nanjing University Center (China) was 132.6% higher than in the normorrhythmic patients [25].

### 4.9. Rational Use of Calcium Score

The determination of the coronary artery calcification index is a valuable method for the cardiovascular risk stratification. It is a helpful screening tool. Depending on the quality of the equipment, the centres performing the tests use different values of the CS coefficient (from 400 according to Agatston), above which the angiographic phase is abandoned. Some cameras provide a high image quality regardless of the intensity of the calcification in the coronary arteries. The information from the calcium score test can be used to modify the angio-CT protocol of the coronary arteries, e.g., to adjust the scanning range [8,19]. The calcium score is not performed in the patients after the coronary angioplasty (PTCA) and coronary artery bypass grafting (CABG). The doses resulting from the calcium score assessment in the coronary vessels can range from 1.7 to 2.6 mSv, according to the ICRP 103 [24].

### 4.10. Multi-Slice, Dual-Source and Wide-Field Tomography

In a retrospective analysis of 278 patients performed in the years from 2015–2017, the DLP values for the examined protocols, including the calcium score and CCTA with the use of the 70–120 kVp tube voltage, were 35.4 mGy*cm (28.3–43.9) for the calcium score, 44.8 mGy*cm (36.6–64.6) for high *pitch factor* helical acquisitions, 94.3 mGy*cm (56.4–175.9) for the sequential, prospective acquisitions and 340.4 mGy*cm (215.6–590.4) for the retrospective spiral acquisitions. For the high-pitch spiral acquisitions, the authors determined the effective dose of 0.63 mSv (0.51–0.90) for the CCTA alone, using an organ conversion factor of k = 0.014 mSv/mGy*cm. The tests were performed using a third-generation dual-source CT system (Somatom Force, *Siemens Healthineers*) [55]. The use of a high pitch factor scanning technique with ECG monitoring reduced the radiation dose by 30% [29,30].

The CONVERGE study compared groups of 110 patients using 64-slice and 256-slice cameras in each group. The determined mean DLP values in the group tested using the 256-slice instrument (*Revolution CT*, *GE Healthcare*) were 113.5 ± 53.6 3 mGy*cm (1.59 ± 0.75 mSv) and were 32% lower than the group tested using the 64-slice instrument for the patients with normal BMI values (18.5–24.9) [56].

In a multicentre study covering 92 patients using scanners equipped with two radiation sources and spectral dual-source photon-counting detector coronary computed tomography angiography (PCD-CCTA) (*Naeotom Alpha*, *Siemens Healthineers*), the mean DLP was 234.1 ± 347.6 mGy*cm and the median DLP was 90.9 mGy*cm (IQR 52.8–235.5 mGy*cm). Using the organ conversion factor k = 0.015 mSv/mGy*cm, the authors determined it to be 1.4 mSv (IQR 0.8–3.5 mSv). The dose depended on the acquisition mode of 1.0 ± 0.8 mSv for the spiral acquisitions with a high *pitch factor*, 4.8 ± 4.0 mSv for the sequential, prospective acquisitions and 9.6 ± 4.4 mSv for the retrospective spiral acquisitions with a low *pitch factor* [57].

## 5. Doses in the TAVI Studies

Transcatheter aortic valve implantation (TAVI) or transcatheter aortic valve replacement (TAVR) was performed for the first time in 2002. The first procedures in Poland were performed in 2008. Initially, this procedure was recommended for patients with severe, symptomatic aortic stenosis who were ineligible for surgery or for whom the surgery was associated with a high risk. Currently, the indications have been extended to patients for whom conventional surgery carries an intermediate risk. In the first period of development for TAVI/TAVR procedures, computed tomography was used to assess the vascular access. Currently, it is also used to accurately image the aortic root with the aortic valve, including determining the dimensions of the aortic annulus, determining the risk of the coronary artery ossification and planning the optimal C-arm angles, which reduces the radiation exposure, the centre volume duration of the procedure and the monitoring of the post-procedural complications, including the prosthetic valve leaflet thickening and possible periarticular leaks. CT has, therefore, become the ‘gold standard’ for planning TAVI/TAVR procedures [58].

CT scanning prior to the TAVI/TAVR procedures required images of the aortic root to be obtained using ECG-monitored acquisitions and images of the vascular accesses from the carotid arteries to the femoral arteries, which did not require synchronisation with the ECG recording. In the construction of the CT protocols before TAVI/TAVR procedures, two methods for obtaining the image data sets were used.

**Figure 4:**
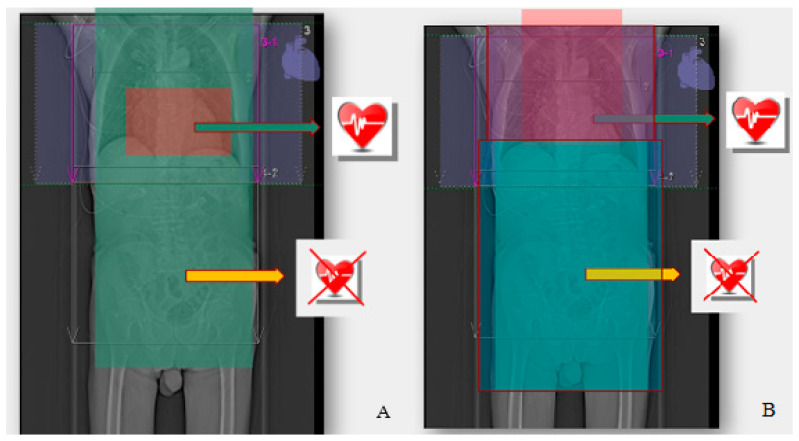
(**A**) ECG-gated acquisition covering the region of the heart and aortic root, followed by the acquisition without ECG gating, covering the vascular accesses in the neck, thorax, abdomen, pelvis and axilla up to the level of the lesser femoral ileum. (**B**) ECG-gated acquisition covering the lower neck and thorax, followed by the acquisition without ECG gating covering the abdomen, pelvis and groin to the level of the lesser femoral ileum.

(1) ECG-gated acquisition covering the region of the heart and aortic root followed by the acquisition without ECG gating covering the vascular accesses in the neck, thorax, abdomen, pelvis and axilla up to the level of the lesser femoral ileum. The disadvantage of this solution was the double acquisition in the area of the aortic root and the heart, which increases the radiation dose. The advantage here, however, was the fact that the time-consuming acquisition of the data synchronised with ECG was reduced to a minimum. By shortening the examination time, the volume of the administered contrast agent could be reduced. It should be remembered that these studies concerned elderly patients, in whom kidney function was most often—even significantly—reduced. In addition, limiting the scope of the ECG-monitored acquisition reduced the part of the examination that required a high dose of radiation, even though the scanning ranges partially overlapped [58], as shown in Figure 4A.

(2) ECG-gated acquisition covering the lower neck and thorax followed by the acquisition without ECG gating covering the abdomen, pelvis and groin to the level of the lesser femoral ileum. The disadvantage of this solution was a higher radiation dose and extended acquisition time for the entire chest. An extended acquisition time may require a larger volume of contrast for the medium and longer breath hold times, which may result in respiratory artifacts in the less resilient patients [58], as shown in Figure 4B.

In order to optimise the radiation dose, the authors of the SSCT guidelines noted that for the patients with a body weight of up to 90 kg or BMI values up to 30 kg/m^2^, the acquisition should use a tube voltage of 100 kVp, and for the patients with a body weight > 90 kg or BMI values > 30 kg/m^2^, the acquisition should use a tube voltage 120 kVp. If the parameters of the scanner allow it, for the patients with BMI values < 26 kg/m^2^, the acquisition should be carried out with an 80 kVp voltage of the apparatus tube [58].

In a national survey conducted in 2018 in Great Britain, 47 responses (12% response rate) were obtained from 40 cardiology centres. A total of 23 centres (58%) also performed TAVI procedures. Most centres (27–59%) performed less than 100 examinations per month. The acquisition protocols varied, where 41% of centres performed retrospective acquisitions with an ECG-monitored radiation dose modulation, 47% performed prospective acquisitions with ECG gating with narrow padding and 12% performed prospective acquisitions with ECG gating and wide padding. The median dose-length product was 675 mGy*cm (IQR 477–954 mGy*cm). The median DLP in the prospective protocols with ECG gating with narrow padding was 423 mGy*cm. The use of a wide padding (30–80% R-R spacing) more than doubled the DLP to 921 mGy*cm. The retrospective acquisition was associated with a high median DLP of 882 mGy*cm [32].

In a study conducted at the Charité University Hospital (Berlin), two study protocols performed on an 80-slice Aquillion Prime instrument (Canon Medical Systems, Otawara, Japan) were compared. In the first of the protocols, the acquisition covered the section of the chest from the top of the lungs to the aortic arch without ECG gating, then the section from the aortic arch to the diaphragm with ECG gating, and finally, the section from the diaphragm to the groin without ECG gating. In the second protocol, the acquisition was carried out uniformly from the lung peaks to the axillae without ECG monitoring with a high *pitch factor* = 1.388). The mean DLP was 790.90 ± 238.15 mGy*cm for the protocol with ECG gating compared to 357.10 ± 200.25 mGy*cm in the high pitch factor protocol without ECG monitoring. The mean effective doses were 13.44 ± 4.05 mSv and 6.07 ± 3.40 mSv, respectively, and the mean SSDEs were 13.84 ± 2.94 mGy and 5.69 ± 2.27 mGy, respectively. The high-spike CT protocol without ECG monitoring reduced the radiation exposure by 55% compared to the protocol with ECG monitoring (from 13.44 mSv to 6.07 mSv). The authors reported statistically significant differences in the SNR and CNR values at the aortic root level between the two groups. However, in the subjective assessment of the Likert scale by two radiologists experienced in imaging the cardiovascular system, the quality of the aortic root image did not show any significant differences [59].

In a study involving 30 randomly selected patients who were tested using the Somatom Force scanner (Siemens Healthcare, Erlangen, Germany), the DLP was 217.6 ± 12.1 mGy*cm (range 178–248 mGy*cm). The acquisition was conducted from the level of the aortic root to the axilla, prospectively with ECG triggering and a high pitch factor. The acquisition was triggered at 60% of the R-R interval using a voltage of 100 kVp and a current of 350 mAs/rev [60].

Additionally, the Somatom Force scanner (Siemens Healthcare, Erlangen, Germany) was used to examine 226 patients in 2018–2019. The first stage of the protocol included a calcium score scan of the aortic valve with the use of a 120 kVp voltage from the level of the tracheal bifurcation to the diaphragm. In the second stage, the prospective acquisition was triggered at 30% of the R-R interval with ECG triggering using a high pitch factor = 3.2 and a voltage of 100 kVp. The mean DLP in this study was 201.1 ± 22.7 mGy*cm [61].

In the work covering 115 studies carried out in 2016–2017, the authors reported an average DLP value of 479.1 ± 45.7 mGy*cm. The study was conducted using a 256-slice revolution CT scanner (GE Healthcare, Milwaukee, WI, USA). The study protocol included the thorax from the lung peaks to the diaphragm in two blocks monitored by an ECG. The study was performed at 100 kVp using a retrospective acquisition, with padding at the 500 ms R-R interval, and a spiral acquisition without ECG monitoring from the diaphragm to the proximal third of the thighs [62].

Another protocol was used for 42 patients who were examined using the Somatom Definition Flash device (Siemens Healthcare, Forchheim, Germany). The acquisition of the cardiac structures from the aortic arch to the diaphragm was performed using ECG monitoring at 60% of the R-R interval, followed by craniocaudal scanning from the top of the lungs to the groin without ECG monitoring and spiral acquisition. Depending on the BMI value, the voltages of 100 kVp and 120 kVp were used. The mean DLP value was 241 ± 27 mGy*cm [63].

## 6. The Postulated New Values of The Organ Conversion Factor—k

In current practice, the effective dose conversion factors for cardiac CT scans are assumed to be the same as for conventional chest CT scans. However, some publications have identified that different, organ-specific conversion factors should be used in cardiac CT scans, which are higher than in conventional chest CT scans.

In one study, the commercial ImPACT CT patient dosimetry calculator program (version 1.0, ImPACT 2009) was used to calculate the CTDIvol, the DLP and the effective dose using the tissue weighting factors, according to the ICRP 103 publication for the cardiac CT model in adult patients with a body weight of 70 kg and a tube voltage from 80 to 140 kV for two systems of 64- and 128-slices (General Electric and Siemens Healthineers). The scope of the survey was 16 cm. The maximum E/DLP values were 0.0375 mSv/mGy*cm and were located in the breast region, which is a particularly sensitive organ. The E/DLP values at the apex of the lung were five times lower and amounted to 0.007 mSv/mGy*cm. The conversion factor developed by the authors for the CT scan of the heart with the examination range of 16 cm and a voltage of 120 kV was 0.0264 mSv/mGy*cm, while the chest CT scan with the examination scope of 36 cm was 0.021 mSv/mGy*cm. It was approximately 70% higher than the current k-factor value adopted for chest CT, which was ∼0.014–0.017 mSv/mGy*cm [20].

The CORE-64 study (the coronary artery evaluation using 64-slice multidetector computed tomography angiography study) used the Monte Carlo calculations for the studies from the Aquillion 64-slice scanner. The study was performed at nine centres. The organ dose and the effective dose resulting from the cardiac CT protocol were assessed. Six voxel patient models were used, representing the examined three men and three women with different body constitutions, i.e., small, normal and obese. They were performed at a tube voltage of 120 kV in a protocol consisting of topograms, calcium scores and the CCTA triggered by a bolus of the contrast agent. The breast tissue weighting factor of 0.24 (ICRP 103) was used in the women, but not in the men. Using the ICRP 103 standards, the sex-averaged organ conversion k-factor for calculating the effective dose from the DLP was 0.030 mSv/mGy*cm (range 0.019–0.043 mSv/mGy*cm). The authors of the study noted that the use of the organ conversion k-factor of 0.017 mSv/mGy*cm, adapted for conventional thoracic CT examinations to estimate the effective dose, resulted in an underestimation of the effective dose of 43% if ICRP 103 standards were used [24].

In 2017, the results of the work based on dosimetry using radiation detectors with MOSFET field-effect transistors were published. These detectors were placed in the topography of 27 organs contributing to the determination of the effective dose in anthropomorphic phantoms and scanned using numerous cardiological CT protocols. The doses in the larger or highly radiation-sensitive organs, such as the lungs and breasts in women, were determined on the basis of the measurements in multiple detectors. In total, 41–44 detectors were used. A total of 120 protocols, performed on 12 CT scanners from five manufacturers (GE, Hitachi (Marunouchi, Chiyoda-ku, Tokyo, Japan), Philips, Siemens, Canon), were examined. The study protocol included topograms, calcium scores and the CCTA using a tube voltage of 70–140 kVp and simulating a pulse rate of 60 and 80 bpm. The effective dose measurements in the phantoms and dose-length products reported by the CT scanner were used to determine the k-factors. The organ conversion k-factor was, on average, 0.026 mSv/mGy*cm and ranged from 0.020–0.035 mSv/mGy*cm. The standard k-factor for the conventional chest CT scans underestimated the calculated effective dose by an average of 46%, ranging from 30% to 60%, depending on the scanner, mode and tube voltage of the scanner. The authors of the study identified that the k-factor for the conventional chest CT examinations was not designed for cardiological examinations. It was based on the older (now replaced by the ICRP 130) definition of effective dose according to the ICRP 60 publication and was determined using three single-slice devices, which differed significantly in the technology from the machines currently used for cardiac CT examinations [23].

The authors of these papers concluded that the k-factors for the cardiac CT scans for all the scanners and protocols were higher than the currently used k-factors for the conventional chest CT scans. Therefore, the radiation doses from the cardiac CT scans were significantly and systematically underestimated. The use of new k-factors in cardiac CT scans may provide more precise guidance for determining the benefits and risks of the testing. Other authors also mentioned higher organ conversion k-factors in their publications. [27,29,30]

## 7. Summary

Cardiovascular CT examinations are becoming increasingly common, and their number and range of indications will continue to increase. The doses of ionising radiation associated with these examinations have always been relatively high, but have been decreasing significantly in recent years, as shown by the studies of the daily practice. This is due both to advances in CT scanner technology and an increase in the ability to optimise the radiation doses. It is important to remember that radiation from medical sources ranks first in possible exposures and that radiation doses are cumulative. Attention should be paid to the radiation doses emitted to the patient in each case. However, it should be kept in mind that some studies indicated that the organ conversion k-factor for cardiac examinations needs to be increased. Therefore we may currently be underestimating the radiation doses in these examinations. The reference dose levels may help to evaluate the CT protocols used in each centre.

## Figures and Tables

**Table 1:** Radiation doses in the cardiovascular CT scans—a review of the reports.

Study	Year of the Survey/Publication	Kind of Protocol	Radiation Doses
CORE-64 study	2010 (publication)	CCTA	ED 19 mSv (range: 16–26 mSv) DLP 633.3 mGy*cm (range: 533.3–866.6 mGy*cm) (mean) k = 0.030 mSv/mGy*cm
		CS	ED 1.7–2.6 mSv DLP 56.6–86.6 mGy*cm (mean) k = 0.030 mSv/mGy*cm
University Center in Nanjing (China)	2007–2016 (survey)	CS+CCTA	DLP 661.2 mGy*cm (range: 315.2–940.0 mGy*cm) (median)
	2016 (survey)	CS+CCTA	DLP 268.2 mGy*cm (range: 183.0–393.6 mGy*cm) (median)
German Cardiac CT Registry	2009–2014 (survey)	CCTA	DLP 255 mGy*cm (median) k = 0.014 mSv/mGy*cm
		CS	DLP 40 mGy*cm (median) k = 0.014 mSv/mGy*cm
	2014 (survey)	CCTA	DLP 176 mGy*cm (median) k = 0.014 mSv/mGy*cm
USA multicentre data	2012 (publication)	CCTA	DLP 621–1143 mGy*cm (mean) k = 0.014 mSv/mGy*cm
		CS	DLP 214 mGy*cm (mean) k = 0.014 mSv/mGy*cm
		TROCT	DLP 286–1386 mGy*cm (mean) k = 0.014 mSv/mGy*cm
Saudi Arabia four centres	2020 (publication)	CS+CCTA	DLP 383.8 ± 354 mGy*cm (mean)
CRESCENT	2011–2013 (survey)	CS+CCTA	ED 8.5 mSv DLP 607.1 mGy*cm (mean) k = 0.014 mSv/mGy*cm
		CS	ED 2.4 mSv DLP 171.4 mGy*cm (mean) k = 0.014 mSv/mGy*cm
PROTECTION VI	2017 (survey)	CCTA	195 mGy*cm (range: 110–338 mGy*cm) (median)
		CS+CCTA	252 mGy*cm (range: 154–412 mGy*cm)

CCTA—coronary computed tomography angiography; CS—calcium score; k—organ conversion factor.

**Table 2:** Cardiovascular CT scans—dose reference values (DRLs).

Study	Year of the Survey/Publication	Reference Level
PROTECTION I	2007	DLP 1200 mGy*cm
PROTECTION VI	2017	DLP 400 mGy*cm
DRL in CCTA in Australia	2018	DLP 268 mGy*cm for the CCTA DLP 137 mGy*cm for the CS
DRL in CCTA in France	2013	DLP 370 mGy*cm for the prospective protocols DLP 870 mGy*cm for the retrospective protocols
DRL in CCTA in Saudi Arabia	2016	DLP 393 mGy*cm for the prospective protocols DLP 1057 mGy*cm for the retrospective protocols
DRL in CCTA in Taiwan	2017–2019	DLP 560.1 mGy*cm for the CCTA DLP 39.2 mGy*cm for the CS
Ionising Radiation (Medical Exposure) Regulations UK	2017	DLP 380 mGy*cm for the retrospective acquisitions with an ECG gating
		DLP 170 mGy*cm for the prospective acquisitions without padding

CCTA—coronary computed tomography angiography; CS—calcium score; DRL—dose reference values.

## Data Availability

Not applicable.

## Abbreviations

ACR	American College of Radiology
AEC	Automatic exposure control
AHA	American Heart Association
ALARA	As low as reasonably achievable
BEIR Committee	Biologic Effects of Ionizing Radiation Committee
BMI	Body mass index
CS	Calcium score
CT	Computed tomography
CTA	Coronary tomography angiography
CTCA	Computed tomography coronary angiography
CTDI	Computed tomography dose index
CTDIvol	Volumetric computed tomography dose index
DLP	Dose-length product
DLR	Deep learning reconstruction
DLIR	Deep learning image reconstruction
ED	Effective dose
ERD	Effective radiation dose
FBP	Filtered back projection
ICA	Invasive coronarography angiography
IEC	International Electrotechnical Commission
IR	Iterative reconstruction
LAR	Lifetime attributable risk
LET	Linear energy transfer
MOSFET	Metal-oxide-semiconductor field-effect transistor
RDSR	Radiation dose structured report
SSCT	Society of Cardiovascular Computed Tomography
TAVI	Transcatheter aortic valve implantation
TAVR	Transcatheter aortic valve implantation replacement
UNSCEAR	United Nations Scientific Committee on the Effects of Atomic Radiation
